# Genetic structure of *Plasmodium falciparum* populations across the Honduras-Nicaragua border

**DOI:** 10.1186/1475-2875-12-354

**Published:** 2013-10-04

**Authors:** Nerea Larrañaga, Rosa E Mejía, José I Hormaza, Alberto Montoya, Aida Soto, Gustavo A Fontecha

**Affiliations:** 1 Instituto de Hortofruticultura Subtropical y Mediterránea La Mayora (IHSM-UMA-CSIC), Algarrobo-Costa, Málaga 29750, Spain; 2 National Malaria Laboratory, Health Ministry, Tegucigalpa, Honduras; 3 National Center for Diagnosis and Reference, Health Ministry, Managua, Nicaragua; 4 Panamerican Health Organization, Managua, Nicaragua; 5 Instituto de Investigacion en Microbiologia, Escuela de Microbiologia, UNAH, Tegucigalpa, Honduras

**Keywords:** *Plasmodium falciparum*, Honduras, Nicaragua, Population structure, Genetic diversity, Microsatellites

## Abstract

**Background:**

The Caribbean coast of Central America remains an area of malaria transmission caused by *Plasmodium falciparum* despite the fact that morbidity has been reduced in recent years. Parasite populations in that region show interesting characteristics such as chloroquine susceptibility and low mortality rates. Genetic structure and diversity of *P. falciparum* populations in the Honduras-Nicaragua border were analysed in this study.

**Methods:**

Seven neutral microsatellite loci were analysed in 110 *P. falciparum* isolates from endemic areas of Honduras (n = 77) and Nicaragua (n = 33), mostly from the border region called the Moskitia. Several analyses concerning the genetic diversity, linkage disequilibrium, population structure, molecular variance, and haplotype clustering were conducted.

**Results:**

There was a low level of genetic diversity in *P. falciparum* populations from Honduras and Nicaragua. Expected heterozigosity (*H*_*e*_) results were similarly low for both populations. A moderate differentiation was revealed by the F_ST_ index between both populations, and two putative clusters were defined through a structure analysis. The main cluster grouped most of samples from Honduras and Nicaragua, while the second cluster was smaller and included all the samples from the Siuna community in Nicaragua. This result could partially explain the stronger linkage disequilibrium (LD) in the parasite population from that country. These findings are congruent with the decreasing rates of malaria endemicity in Central America.

## Background

In the region of the Americas, malaria still occurs in 21 countries with about 30% of inhabitants at some risk of infection and 8% at high risk. During 2011, the largest number of cases occurred in countries that share the Amazon rainforest, such as Brazil, Colombia, Peru, and Venezuela, while in the Mesoamerica subregion malaria cases are concentrated in Honduras, Guatemala and Nicaragua, in descending order of importance [1]. In those countries, less than 8% of malaria cases are caused by *Plasmodium falciparum* and the remaining cases are caused by *Plasmodium vivax* (based on data provided by National Health Ministries). Thanks to the tremendous efforts of national and international organizations, a reduction of more than 75% of incidence rates has been confirmed in six countries of this region between 2000 and 2011. The achievements are even greater for Costa Rica and El Salvador, which are now considered in the malaria pre-elimination phase [1].

**Figure 1:**
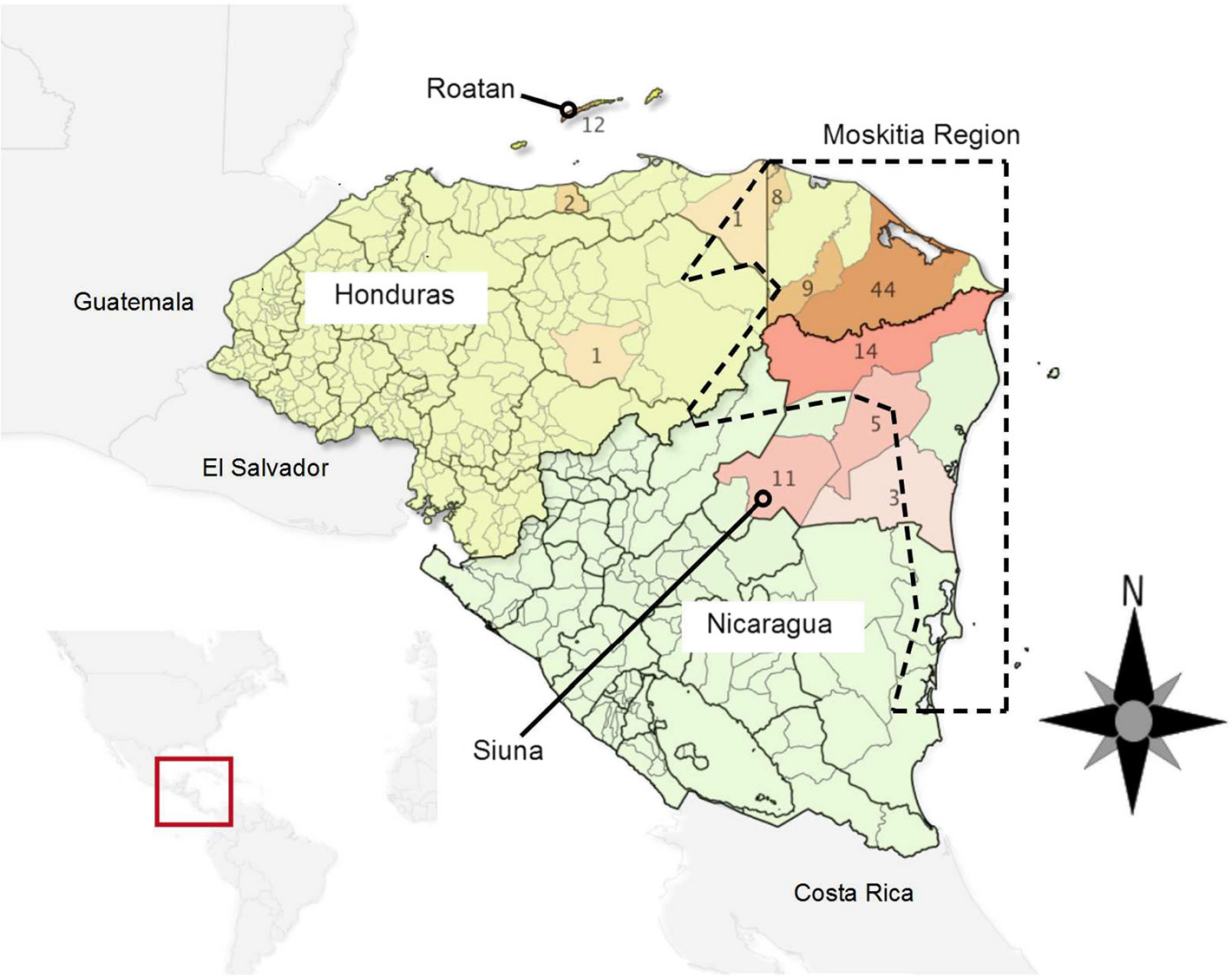
**Partial map of Central America showing collection sites of samples infected by *Plasmodium falciparum. ***Municipalities have been coloured with different shades to indicate the proportion of samples collected in each location. Dot lines indicate approximate Moskitia region limits.

The distribution of malaria cases caused by *P. falciparum* in Central America is limited to the coastal areas of the Caribbean, such as the Bay Islands in Honduras and the region called “Moskitia”. Moskitia is a territory shared by Honduras and Nicaragua (Figure 1), with a tropical rainforest-dominant ecosystem and particular conditions concerning the epidemiology of the disease and social characteristics, such as extreme poverty, social inequity and low educational levels. This region contributes most cases of infections by *P. falciparum* in Central America [2]. In spite of the relative small territory of Central American countries, cultural barriers (the human population of Moskitia is mostly indigenous Miskito, with their own language) and severe access problems tend to isolate Miskito communities from the rest of the society.

One of the most notable epidemiological characteristic of *P. falciparum* populations in Central America is the susceptibility to chloroquine (CQ) [3,4], which ceased to be useful a long time ago in other regions of the world, including South America [5,6]. CQ susceptibility of the pathogen is tremendously advantageous to fight against malaria and this is partly the reason for the recent success in malaria control in Central America. However, there are some environmental and anthropogenic factors that could challenge national malaria control programmes, such as deforestation due to agricultural practices driving an increase in vector abundance, climatic change, or illegal migration of people from south to north through the Central American isthmus.

Genetic diversity indexes can be used as indicative of the adaptation and fitness of *Plasmodium* populations in a particular ecosystem [7]. Extensive analyses of genetic diversity of *Plasmodium* species have been reported worldwide in order to better understand the biology of the parasite [7-9] and the acquisition of immunity to malaria [10]. Those studies have demonstrated how especially useful it is to predict the spreading of phenotypes of interest concerning drug resistance [9,11,12], and target antigens for vaccine development [13-16]. Multilocus genotyping of neutral molecular markers is one of the most frequent approaches used to determine the structure of *P. falciparum* populations. Consensus patterns of population structure have been found depending on the transmission rate in different regions [9,17,18]. In general terms, high genetic diversity, low linkage disequilibrium (LD) and weak population differentiation are typical of high transmission areas [19]. Highly endemic areas, such as sub-Saharan Africa, also show more frequent outcrossing of isolates, leading to panmictic structures of parasites with abundant genotypes. In the Americas, the opposite genetic scenario seems more likely: lower genetic diversity, high levels of LD and well-defined population structure [20].

In the Americas, population analyses of *P. falciparum* using neutral microsatellites or simple sequence repeats (SSR) have been reported from South America. Most of the results confirm that limited genetic diversity, significant LD, and gene flow are common for Brazil, Peru, Colombia, and Bolivia parasite populations [5,17,20-23]. In Central America, a limited number of studies on molecular techniques [24], drug resistance [3,4,25] and genetic diversity based on conserved genes [13,26] of *P. falciparum* are available, and mostly from Honduras. However, genetic analyses of populations of this parasite based on neutral microsatellites in Central America have not been reported so far. Taking into account recent changes in the demography and epidemiology of malaria in this region, in this work the population structure and genetic diversity of *P. falciparum* was assessed in 11 municipalities from Honduras and Nicaragua using seven SSR neutral markers.

## Methods

### Parasite samples

One-hundred and ten human blood samples from Honduras and Nicaragua areas with endemic malaria falciparum transmission were analysed. Eighty-three (75.45%) of those samples were taken from patients living in the Moskitia region. Seventy-seven samples were collected from seven municipalities of Honduras, and 33 from four municipalities of Nicaragua (Figure 1). Twelve of 77 samples from Honduras were collected in the Caribbean island of Roatan while the rest of samples were collected in the continental territory. In Honduras, samples were collected between 2009 and 2012 in national health care centres after informed consent from patients that sought medical attention. Scientific approval and ethical clearance was obtained from the Ethics Board Committee of the Hearth-Lung National Institute, Ministry of Health of Honduras. In Nicaragua, samples were collected from patients attending routine medical diagnosis of malaria, with no initial intent to be used in research. Samples were processed without personal identifiers or hospital record numbers to avoid the risk of future re-identification of individuals. The PAHO local Office at Nicaragua and National health authorities approved this process.

All patients showed symptoms typical of malaria at the time of enrolment. Filter paper was used to collect one drop of blood from each patient for malaria surveillance purposes. Diagnosis of *P. falciparum* infections were carried out by experienced microscopists, and molecular confirmation of the species was obtained through PCR amplification of the 18Sr gene according to Singh *et al*. [27]. Three *P. falciparum* isolates from sub-Saharan Africa and five universally known reference clones (3D7, Dd2, HB3, 7G8 and K1) were included in this study as an outgroup and amplification controls.

### Microsatellite genotyping

DNA from *P. falciparum*-positive filter paper blood spots was extracted using a Chelex-based method [28]. The following neutral SSR loci were amplified (chromosome location in parentheses) according to previously described protocols: TA1 (6), Poly-α (4), PfPK2 (12), TA109 (6), 2490 (10), [17], C2M34 (2) and C3M69 (2) [12]. Forward primers were labelled with a fluorescent dye on the 5′-end and PCR products were detected and size measured with a Beckman Coulter CEQ™ 8000 capillary DNA analysis system. Samples were denaturalized at 90°C for 120 seconds, injected at 2.0 kV, 30 seconds, and separated at 6.0 kV for 35 min. Water was used as negative control against contamination in each run and each PCR reaction was repeated at least twice to ensure the reproducibility of the results. Since *Plasmodium* parasites are haploid organisms during most of their life cycle, except for a brief period after fertilization inside the gut of the female *Anopheles*, each allele obtained in the pherogram would represent a different clone of the parasite and in single clone infections, one allele would be expected per locus in each samples. In those cases when a secondary peak was observed in the chromatogram, the height of the secondary peak was measured. If the height of the minor peak was lower than 30% of the predominant peak they were considered as single clone infections [7] and, consequently, all the calculations were performed taking into account only the predominant allele (more intense PCR product) when two peaks were present.

### Data analysis

Genetic diversity parameters were calculated for the entire population and for two subpopulations (Honduras and Nicaragua) independently using ARLEQUIN version 3.5 software [29] by determining the number of alleles per locus (*A*), allelic richness (*R*_*s*_), expected heterozigosity (*H*_*e*_), allele frequencies and the number of haplotypes (*h*). Three of these parameters (*A*, *H*_*e *_and allele frequencies) were used to indicate the level of polymorphism in the loci and determine the diversity of the populations. *H*_*e *_was calculated as $\mathrm{He}=\frac{n}{n-1}\left(1-\sum_{i=1}^{n}P_{i}^{2}\right)$, where *n* is the number of isolates and *ρ*_*i *_is the frequency of *i*^th^ allele.

Since *A* is strongly dependent on sample size and different number of isolates were analysed in the two countries (77 in Honduras *vs* 33 in Nicaragua), it was necessary to normalize the data. For that, the allelic richness (*R*_*s*_) was calculated using FSTAT version 2.9.3 software, fixed as the smallest number of individuals typed for a locus in a sample.

The ARLEQUIN software was also used to estimate population differentiation using the Wright’s pair-wise fixation index *F*_*ST*_, which is a pair-wise distance measurement used to calculate the average F statistics over all loci according to the number of different alleles between haplotypes [30].

Multilocus linkage disequilibrium (LD = non-random association of alleles among loci) was measured using the Standardized Index of Association ($I_{A}^{S}$); both $I_{A}^{S}$ and variance of data were calculated using the program LIAN version 3.5 [31]. $I_{A}^{S}$ was calculated as follows: (*V*_*D*_ / *V*_*e*_ - 1)/(n - 1) assuming a null hypothesis of complete linkage equilibrium ($I_{A}^{S}=0$), where V_D_ is the variance of the number of alleles shared between all pairs of haplotypes observed in the population, V_e_ is the variance expected under linkage equilibrium, and n is the number of loci examined.

To establish the structure of the populations according to geographic origin, Structure version 2.3.3 software was used. This software assigns probable individual haplotypes to multiple clusters (*K*) relying on the allele frequencies at each locus, assuming that within populations, the loci are at Hardy-Weinberg equilibrium, and linkage equilibrium [32]. An admixture option was used. To choose the best value of *K*, five independent replicates were run for 100,000 steps, after a burn-in period of 10,000 steps, and the mean log likelihoods in each run were compared. The most likely number of populations was calculated by the higher number in the rate of change for *K* (*ΔK*) [33]. The structure of *P. falciparum* subpopulations was confirmed by analysis of molecular variance (AMOVA) [34]. Similarity between haplotypes was examined by creating a similarity matrix with Dice’s coefficient to construct a UPGMA (Unweighted pair group method with arithmetic mean) dendrogram using NTSYSpc version 2.11f software [35].

## Results and discussion

This study included 110 *P. falciparum* isolates from 11 communities located in Honduras and Nicaragua, mostly from the Moskitia region across the border between both countries (Figure 1). This region is characterized by a low to moderate rate of malaria transmission caused by *P. falciparum* compared to other geographic regions such as Africa or Southeast Asia. Seven neutral microsatellite loci located on five chromosomes and widely scattered in the genome of *P. falciparum* were analysed. These and other SSR markers have been used to analyse the genetic structure of *P. falciparum* populations from almost every malarial region in the world, including Africa [17,36,37], Southeast Asia [8,18,38,39], Oceania [7], and South America [5,20,21]. However, this is the first reported study of the genetic structure of *P. falciparum* involving analysis of microsatellite loci in isolates from the Moskitia region in Central America and the comparison of population structure between parasites from Honduras and Nicaragua.

### Genetic diversity

Of the 110 isolates, data for all seven loci were obtained for 88 (80%). For 16 (14.5%) isolates allelic data were obtained for 6 loci; and the remaining 6 (5.5%) isolates failed to amplify 2 loci. Most of the isolates analysed (99.6%), revealed one single peak in the chromatogram but three isolates from Honduras showed two peaks for one locus each. A full description of allele sizes, missing data and mixed-clone infections is given as Additional file 1.

Number and frequencies of SSR alleles were calculated (Table 1). Allele sizes reported in this study ranged within the expected according to most of previous reports with two exceptions: Poly-α and TA109 loci showed much longer allele sizes in isolates from Peru [40] and Kenya [41], respectively. The number of alleles per locus in the total population was low and varied from one (TA1) to five alleles (Poly-α, C3M69) (Table 1) ranging from two to five alleles in the samples taken in Honduras and from one to four in the samples taken in Nicaragua. There were no significant differences in the mean number of alleles per SSR locus between the samples taken from those two countries (Table 2) calculated by a *t* test. Normalized allelic richness (*R*_*S*_) showed a similar pattern, ranging from 1.83 to 4.08 in Honduras, and from 1 to 3.98 in Nicaragua.

**Table 1:** Allelic frequencies at seven microsatellite loci

**Locus**	**Allele**	**Honduras**	**Frequency**	**Nicaragua**	**Frequency**
		**(n = 77)**		**(n = 33)**	
TA1	141	61	0.79	19	0.58
	144	11	0.14	-	-
	147	1	0.01	1	0.03
	169	-	-	13	0.39
	190	3	0.04	-	-
	MD	1	0.01	-	-
POLY- α	141	3	0.04	13	0.39
	169	1	0.01	-	-
	173	6	0.08	-	-
	179	66	0.86	20	0.61
	181	1	0.01	-	-
PFPK2	131	1	0.01	-	-
	162	24	0.31	3	0.09
	189	7	0.09	12	0.36
	195	45	0.58	17	0.52
	MD	-	-	1	0.03
TA109	189	49	0.64	24	0.73
	201	25	0.32	8	0.24
	MD	3	0.04	1	0.03
2490	80	2	0.03	-	-
	83	74	0.96	33	1
C2M34	225	55	0.71	27	0.76
	227	9	0.12	-	-
	MD	13	0.17	6	0.08
C3M69	125	20	0.26	9	0.27
	137	8	0.10	1	0.03
	139	44	0.57	9	0.27
	141	-	-	13	0.40
	MD	5	0.06	1	0.03

MD = missing data.

**Table 2:** **Allelic diversity at seven microsatellite loci of *Plasmodium falciparum *in Honduras and Nicaragua**

**Locus**	A* ( R_s_**)		**Expected heterozygosity (H**_**e**_**) (s.d.)**			
	**HN (n = 77)**	**NIC (n = 33)**	**HN**	**NIC**	**Mean (s.d.)**	**Tot. Het.**
TA1	4 (3.52)	3 (2.97)	0.34	0.53	0.43 (0.14)	0.44
POLY-A	5 (4.08)	2 (2)	0.26	0.49	0.38 (0.16)	0.37
PFPK2	4 (3.58)	3 (3)	0.56	0.59	0.57 (0.02)	0.59
TA109	2 (2)	2 (2)	0.45	0.39	0.42 (0.05)	0.43
2490	2 (1.83)	1 (1)	0.05	0	0.03 (0.04)	0.04
C2M34	2 (2)	1 (1)	0.25	0	0.12 (0.17)	0.18
C3M69	3 (3)	4 (3.98)	0.54	0.70	0.62 (0.11)	0.65
**Mean**	**3.14 (2.86)**	**2.29 (2.28)**	**0.35 (0.18)**	**0.38 (0.28)**	**0.37 (0.02)**	**0.38**
**s.d.**	**1.15 (0.91)**	**1.11 (1.10)**				

*A = Numbers of different alleles within each population.

** R_S_ = allelic richness.

The number of exclusive alleles observed in only one of the two countries (Honduras *vs* Nicaragua) ranged from zero (for TA109 locus) to three (Poly-α). In general terms, the number of alleles per locus tends to be higher in areas of high endemicity, and lower in areas of low or moderate endemicity [18,40]. In this case, because of its low number of alleles, the Moskitia region could be comparable to regions of similar epidemiology, such as Amazonia [17], Malaysia [18] and Philippines [42] with an unstable pattern of malaria transmission [26], and where allele diversity remains almost unchanged in time [43]. A different scenario can be observed in regions with high endemicity such as Sub-Saharan Africa, with allele numbers ranging from 5.3 [44] to 13.5 [37] for similar loci.

The small variation in allele numbers between countries was further evaluated by expected heterozigosity (H_e_). H_e_ was similar for both populations in Honduras (0.35 ± 0.18), and Nicaragua (0.38 ± 0.28) (Table 2), in spite of the difference in the number of samples (77 *vs* 33, respectively).

Low estimates of expected heterozygosities found for Central American *P. falciparum* populations are comparable with other studies based on neutral microsatellite loci in South America: Brazil (0.14-0.62) [39,45], and Peru (0.34-0.62) [40]. *H*_*e *_values revealed to be lower than those found in other countries with higher endemicity, such as: Vietnam (0.52-0.91), three West African countries (0.78-0.96) [46], Nigeria (0.83-0.92), Sudan (0.67-0.90) and South Africa (0.76-0.96) [45]. This result also indicates that heterozygosity levels in Central American populations are compatible with those expected in areas of a proportional lower malaria transmission with no frequent outcrossing.

### Population structure

The F_ST_ coefficient was estimated for both subpopulations using seven microsatellite loci. F_ST_ is based on the differences in allele frequencies in each population. The number of isolates used to calculate F_ST_ between Honduras and Nicaragua was 77 and 33, respectively. Values close to zero suggest no frequency differences, and values similar to 1.0 indicate completely fixed differences without shared alleles. F_ST_ index measuring Honduras-Nicaragua inter-population variance in allele frequencies was 0.14. A qualitative interpretation of F_ST_ values has been suggested [47], where values between 0–0.05 could indicate low genetic differentiation among populations; values between 0.05-0.15 could indicate moderate differentiation; values ranging from 0.15-0.25 could indicate great differentiation; and values higher than 0.25 could indicate very great differentiation. Based on this not universally accepted classification, the Honduras parasite population would differ moderately from the Nicaragua population. A recent publication has described the genetic diversity of *P. falciparum* populations in Malaysia through a deep sequencing approach [9]. These authors show that the fixation indexes and parasite population structure tend to increase where the malaria transmission is low or patchy. That patchy population pattern could be occurring in Nicaragua, where the malaria transmission is descending faster than it does in Honduras.

**Figure 2:**
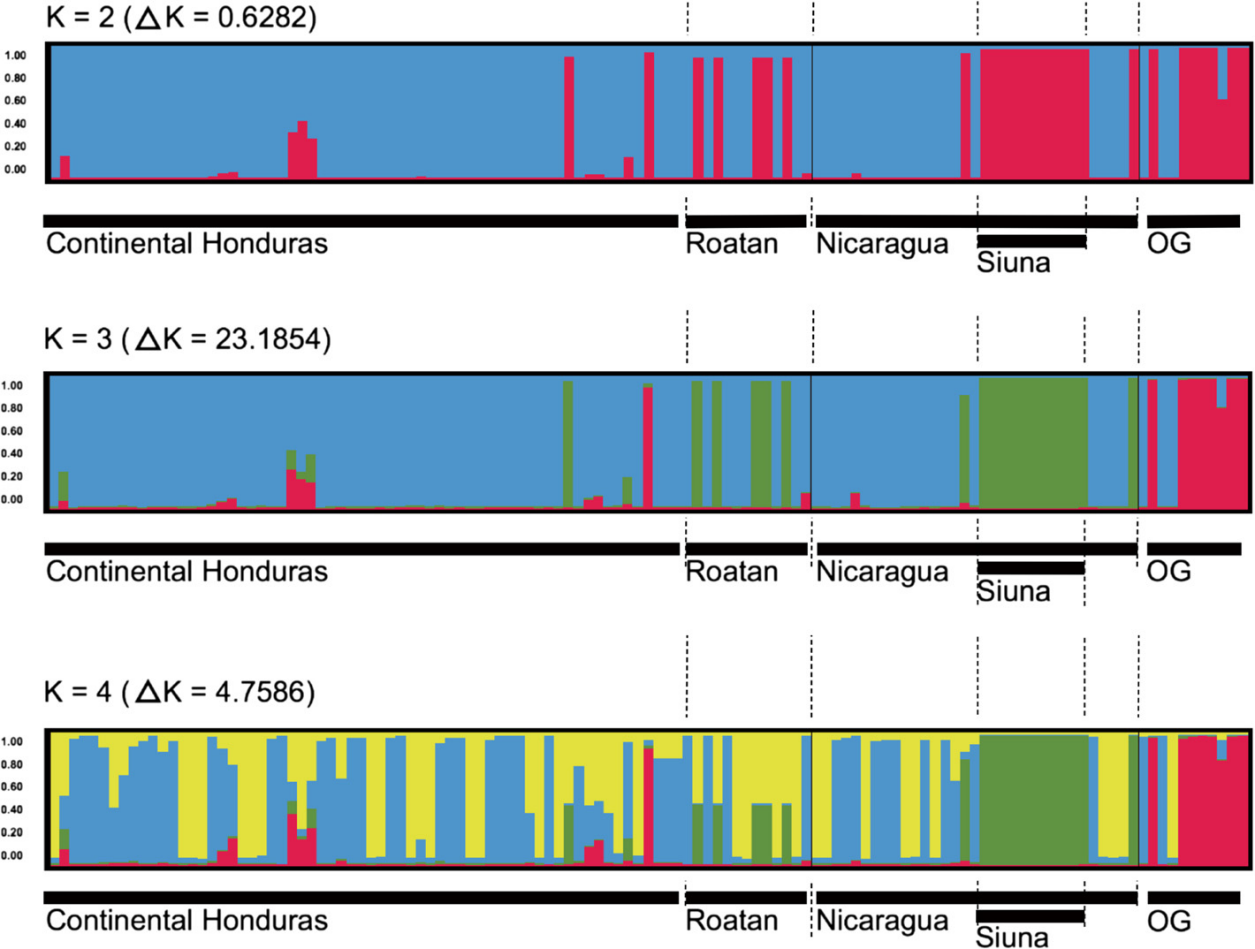
**Bayesian clustering of 110 isolates assigned to K populations based on seven SSR markers**. Each bar represents the proportion of each haplotype with ancestry in the defined clusters, each cluster being indicated by a different colour. OG: outgroup of reference clones parasites with an African origin.

In order to find out the reason for the moderate differentiation between subpopulations, a clustering approach was undertaken using the Structure software. This Software uses a Bayesian analysis to infer the most likely number of populations (*K*), and measures the probability that individual parasites belong to each of these *K* populations [32]. This analysis defined three putative clusters (*ΔK* = 23.19) (Figure 2). The first cluster included most of samples from both countries (Honduras and Nicaragua). The second cluster grouped all the 11 isolates collected in the Nicaraguan community of Siuna, five isolates from Roatan Island, one isolate from Wampusirpi (Honduran Moskitia), one from Rosita, and one from Prinzapolka (Nicaraguan Moskitia). The third cluster the three outgroup isolates of African origin and *P. falciparum* reference clones. This analysis indicates a weak population structure in parasites isolated from continental Honduras and Nicaragua, with the exception of samples grouped into the second cluster. Therefore, Siuna isolates could explain the moderate differentiation between Honduras and Nicaragua samples. Although Siuna is part of the municipalities located in the Nicaraguan region called RAAN (Autonomous Region of North Atlantic) with the highest malaria incidence in Nicaragua, its human population is ethnically different from the inhabitants of the neighbor region of Moskitia and belong to different language cultures. This social difference and the almost inexistent access roads between municipalities could indicate historical separation of human communities and, as a consequence, barriers for gene flow and parasite migration, as suggested by similar studies in other regions [7].

**Figure 3:**
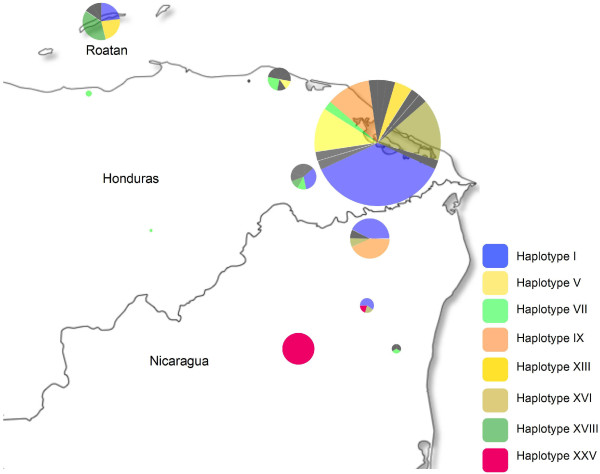
**Haplotype distribution by geographic region.** Less common haplotypes are shaded in grey. Circle sizes indicate the number of isolates collected in those communities.

Confirming this, a haplotype (*h*) analysis defined 25 haplotypes in the whole population (Table 3). Only two haplotypes did not include isolates from Honduras, whilst 17 haplotypes lacked representatives from Nicaragua, which could be a consequence of a lower number of samples from this country. It is noteworthy that haplotype I was the most frequent in both countries: 29% in Honduras and 27% in Nicaragua, and six haplotypes included isolates from both countries. Based on this evidence it is possible to suggest that most parasite haplotypes circulating through Honduras and Nicaragua are not geographically separated in spite of the political border. Once again, the most interesting finding was the exclusive presence of haplotype XXV in the Siuna region with 11 sampled individuals (Figure 3), which could be explained by different, non-mutually exclusive reasons such as an inbreeding phenomenon, a transient clonal expansion of a founder infection.

**Table 3:** **Haplotypes frequency estimation of *Plasmodium falciparum *in Honduras and Nicaragua**

**Haplotype**	**Honduras (n = 77)**		**Nicaragua (n = 33)**	
	**Number of samples**	**Frequency**	**Number of samples**	**Frequency**
I	22	0.29	9	0.27
II	2	0.03	0	0
III	1	0.01	1	0.03
IV	2	0.03	0	0
V	6	0.08	0	0
VI	1	0.01	0	0
VII	7	0.09	1	0.03
VIII	1	0.01	0	0
IX	5	0.06	6	0.18
X	1	0.01	0	0
XI	1	0.01	0	0
XII	1	0.01	0	0
XIII	5	0.06	0	0
XIV	1	0.01	0	0
XV	1	0.01	0	0
XVI	7	0.09	2	0.06
XVII	1	0.01	0	0
XVIII	6	0.08	0	0
XIX	1	0.01	0	0
XX	2	0.03	0	0
XXI	1	0.01	0	0
XXII	1	0.01	0	0
XXIII	1	0.01	1	0.03
XXIV	0	0	1	0.03
XXV	0	0	12	0.36

**Figure 4:**
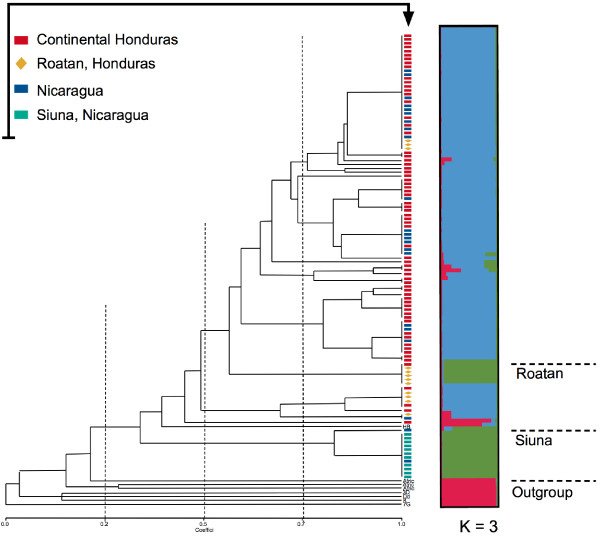
**UPGMA dendrogram showing the relationships between *Plasmodium falciparum *isolates and a putative population structure.** Isolates are coloured according to geographic location in the dendrogram.

A UPGMA dendrogram was constructed to further evaluate similarity between subpopulations. The tree obtained supports the Bayesian clustering pattern shown before. Figure 4 illustrates how isolates from continental Honduras and Nicaragua form an interspersed and poorly differentiated pool of branches. However, the outgroup genotypes and the Siuna population separated into two well-differentiated clusters. A coloured diagram of Structure results (for three populations) with an identical ordering of isolates has been placed on the right side of the dendrogram, aiming to better visualize the population structure. Interestingly, some individuals belonging to the population of Roatan Island reveal a moderately strong cluster placed in the same population as the Siuna samples by the Structure result.

Furthermore, an analysis of molecular variance (AMOVA) was used to assess the population structure of *P. falciparum* in Central America by partitioning variation among and within the Honduras and Nicaragua subpopulations. Results of AMOVA indicated that most of the genetic variation among parasites is contained within populations (86%), and only 14% of variation was explained by comparing subpopulations, suggesting a relative free gene flow across the region. This variation of 14% is consistent with the F_ST_ index between populations described before. Similar results were reported for South American parasite populations, where most of the diversity was also found within locations [23]. In addition, a second AMOVA analysis comparing the two populations described by the Structure software was carried out. In this case, the results showed high differentiation (F_ST_ = 0.56) between both populations, where 55.64% of the variance was explained between populations and 44.36% within populations.

### Multilocus linkage disequilibrium

Existence of multilocus linkage disequilibrium (LD) was calculated for the entire population as a whole, and separately for each subpopulation using the standardized index of association ($I_{S}^{A}$). The Nicaragua parasite subpopulation showed a significantly higher LD value than the Honduran subpopulation (Table 4). In general terms a stronger LD corresponds to low endemicity and *vice versa *[8,17]. In this case, the stronger LD revealed in the Nicaraguan subpopulation could be due to a lower transmission intensity where mixed-clone infections were not found and recombination between parasite genomes is less probable [48,49]. A higher LD value of parasites from Nicaragua, added to an exclusive haplotype in Siuna, suggests a high level of ongoing inbreeding in that population.

**Table 4:** **Linkage desequilibrium analysis for each *Plasmodium falciparum *population**

**Population**	**V**_**D**_	**V**_**e**_	$I_{A}^{S}$	**Var(V**_**D**_**)**	**Unique haplotypes only**	
					**Isolates (n)**	$I_{A}^{S}$
Honduras and Nicaragua	2.4	1.5	0.1051	0.008	-	-
Honduras	1.9	1.5	0.0483	0.012	34	0.072
Nicaragua	3.6	1.4	0.2623	0.009	13	0.088

V_D_ = variance; V_e_ = expected variance if linkage equilibrium exist; $I_{A}^{S}$ = Standardized Index of Association; Var(V_D_) = null hypothesis; V_D_ = V_e_.

A second LD test was also performed but including unique haplotypes for each population only (Table 4). In the parasite populations from Honduras LD maintained similar (0.0483 *vs* 0.0716), however this was not the case for Nicaraguan isolates including 11 isolates from Siuna. LD was considerably reduced from 0.2623 to 0.0886 when only unique haplotypes from Nicaragua were analyzed. This result reinforces the hypothesis that only a small population of parasites from Nicaragua behaves as a residual focus in an area of declining endemicity.

## Conclusions

Genetic diversity indexes and population structure of *P. falciparum* were determined in Honduras and Nicaragua using seven SSR neutral loci. This approach is an important tool to better understand the epidemiologic factors that need to be focused in order to control infections and to anticipate emergence of drug resistance. Honduras and Nicaragua are two of the few remaining countries in the world where *P. falciparum* and *P. vivax* are still susceptible to CQ and sulphadoxine-pyrimethamine [3,4]. This phenomenon has not been clearly explained so far, but several authors have theorized about a possible correlation between population structure and emergence/spread of drug resistance in different regions [18,42,50]. This study has demonstrated gene flow and lack of significant genetic structure in *P. falciparum* populations between Honduras and Nicaragua when neutral markers are analysed. In consequence, those results could contribute to the untangling of the reasons for drug susceptibility and the potential for drug-resistant mutation emergence in Central America during malaria control and elimination stages.

## Abbreviations

Dd2: *Plasmodium falciparum* strain collected in Indochina/Laos; HB3: *Plasmodium falciparum* strain collected in Honduras; 7G8: *Plasmodium falciparum* strain collected in Brazil; K1: *Plasmodium falciparum* strain collected in Thailand; 3D7: *Plasmodium falciparum* clone from NF54, which was isolated from a patient who lived near the airport in Amsterdam, the Netherlands. The origin of the infection is unknown.

## Competing interests

The authors declare that they have no competing interests.

## Authors’ contributions

GF and NL carried out the DNA extraction and performed molecular experiments. GF, NL and IH drafted the manuscript and all authors read and edited the manuscript. RM, AM, and AS participated in sample collection and microscopic analyses coordination. GF, NL, and IH conceived the study and participated in its design and coordination and helped to draft the manuscript. All authors read and approved the final manuscript.

## Supplementary Material

Additional file 1 **Microsatellite allele sizes for *Plasmodium falciparum *populations from Honduras, Nicaragua and reference clones.** Click here for file
