# Supplementary material for: Genetic structure of Plasmodium falciparum populations across the Honduras-Nicaragua border
Source: Malar J. 2013 Oct 4;12:354. doi: 10.1186/1475-2875-12-354 (PMC3851272; doi:10.1186/1475-2875-12-354)
Supplement: Additional file 1 — Microsatellite allele sizes for Plasmodium falciparum populations from Honduras, Nicaragua and reference clones. [file 1475-2875-12-354-S1.pdf]

N	Code	TA1 (bp)	POLYA (bp)	PFPK2 (bp)	TA109 (bp)	2490 (bp)	C3M69 (bp)	C2M34 (bp)
1	HND1	**144**	**179**	**162**	**189**	**83**	**125**	**225**
2	HND2	**141**	**179**	**195**	**189**	**83**	**137**	**225**
3	HND3	**141**	**179**	**195**	**201**	**83**	**139**	**227**
4	HND4	**141**	**179**	**195**	**201**	**83**	**125**	**225**
5	HND5	**141**	**179**	**195**	**189**	**83**	**139**	**225**
6	HND6	**141**	**179**	**195**	**189**	**83**	**139**	**225**
7	HND7	**141**	**179**	**195**	**189**	**83**	**139**	**225**
8	HND8	**141**	**179**	**195**	**189**	**83**	**139**	**225**
9	HND9	**144**	**179**	**162**	**189**	**83**		
10	HND10	**141**	**179**	**162**	**201**	**83**	**137**	**227**
11	HND11	**141**	**179**	**195**	**189**	**80**	**125**	**225**
12	HND12	**144**	**179**	**195**	**189**	**80**	**139**	**225**
13	HND13	**141**	**179**	**195**	**189**	**83**	**139**	**225**
14	HND14	**141**	**179**	**195**	**189**	**83**	**139**	**225**
15	HND15	**141**	**179**	**195**	**201**	**83**	**139**	**227**
16	HND16	**141**	**179**	**195**	**201**	**83**	**139**	**227**
17	HND17	**141**	**169, 173**	**189**	**189**	**83**	**139**	**225**
18	HND18	**141**	**173**	**189**	**189**	**83**	**139**	**225**
19	HND19	**141**	**179**	**162**	**201**	**83**	**137**	**227**
20	HND20	**141**	**173**	**189**	**189**	**83**	**139**	**225**
21	HND21	**141**	**179**	**162**	**201**	**83**	**137**	**227**
22	HND22	**141**	**179**	**195**	**189**	**83**	**139**	**225**
23	HND23	**141**	**179**	**195**	**189**	**83**	**139**	**225**
24	HND24	**141**	**173**	**189**	**189**	**83**	**139**	**225**
25	HND25	**141**	**173**	**189**	**189**	**83**	**139**	**225**
26	HND26	**141**	**179**	**195**	**189**	**83**	**139**	**225**
27	HND27	**141**	**173**	**189**	**189**	**83**	**139**	**225**
28	HND28	**141**	**179**	**195**	**189**	**83**	**139**	**225**
29	HND29	**141**	**179**	**195**	**189**	**83**	**139**	
30	HND30	**141**	**179**	**195**	**201**	**83**	**125**	
31	HND31	**141**	**179**	**162**	**201**	**83**	**137**	**227**
32	HND32	**190**	**141**	**195**	**201**	**83**	**139**	**225**
33	HND33	**141**	**181**	**195**	**189**	**83**	**125, 139**	**225**
34	HND34		**141**	**195**	**201**	**83**	**139**	
35	HND35	**141**	**179**	**195**	**201**	**83**	**125**	**225**
36	HND36	**141**	**179**	**162**	**189**	**83**	**125**	**225**
37	HND37	**144**	**179**	**195**	**189**	**83**	**139**	**225**
38	HND38	**141**	**179**	**162**	**189**	**83**	**125**	**225**
39	HND39	**141**	**179**	**162**	**189**	**83**	**125**	**225**
40	HND40	**141**	**179**	**195**	**189**	**83**	**139**	**225**
41	HND41	**141**	**179**	**195**	**189**	**83**	**139**	
42	HND42	**141**	**179**	**162**	**189**	**83**	**125**	**225**
43	HND43	**144**	**179**	**162**	**189**	**83**	**125**	**225**
44	HND44	**141**	**179**	**195**	**189**	**83**	**139**	**225**
45	HND45	**141**	**179**	**195**	**189**	**83**		

46	HND46	**147**	**179**	**162**	**201**	**83**	**137**	**225**
47	HND47	**141**	**179**	**162**	**189**	**83**	**139**	**225**
48	HND48	**190**	**179**	**195**	**201**	**83**	**139**	**225**
49	HND49	**190**	**179**	**195**	**201**	**83**	**139**	
50	HND50	**141**	**179**	**195**	**201**	**83**	**139**	**225**
51	HND51	**141**	**179**	**195**			**139**	**225**
52	HND52	**141, 190**	**179**	**189**	**201**	**83**	**125**	**225**
53	HND53	**141**	**179**	**195**	**189**	**83**	**139**	
54	HND54	**141**	**179**	**195**	**189**	**83**	**139**	**225**
55	HND55	**141**	**179**	**162**	**189**	**83**		**225**
56	HND56	**141**	**179**	**162**	**189**	**83**	**125**	**225**
57	HND57	**141**	**179**	**162**	**189**	**83**	**125**	**225**
58	HND58	**141**	**141**	**195**	**201**	**83**	**139**	**225**
59	HND59	**141**	**179**	**195**	**189**	**83**	**125**	**227**
60	HND60	**144**	**179**	**162**	**189**	**83**	**137**	**225**
61	HND61	**144**	**179**	**162**	**189**	**83**	**125**	
62	HND62	**141**	**179**	**162**	**201**	**83**	**139**	**225**
63	HND63	**141**	**179**	**195**	**201**	**83**	**139**	
64	HND64	**141**	**179**	**195**	**201**	**83**		
65	HND65	**144**	**179**	**162**	**189**	**83**	**139**	**225**
66	HND66	**141**	**179**	**195**	**189**	**83**	**139**	**225**
67	HND67	**141**	**179**	**195**	**189**	**83**	**139**	**225**
68	HND68	**141**	**179**	**195**	**201**	**83**	**125**	
69	HND69	**144**	**179**	**162**	**189**	**83**	**125**	**225**
70	HND70	**144**	**179**	**162**	**189**	**83**	**125**	**225**
71	HND71	**144**	**179**	**162**	**-1**	**83**	**125**	**225**
72	HND72	**141**	**179**	**195**	**201**	**83**	**139**	**225**
73	HND73	**141**	**179**	**195**	**189**	**83**	**139**	**225**
74	HND74	**141**	**179**	**162**	**201**	**83**	**137**	**227**
75	HND75	**141**	**179**	**195**	**189**	**83**	**139**	**225**
76	HND76	**144**	**179**	**195**	**189**	**83**	**125**	**225**
77	HND77	**141**	**179**	**195**	**201**	**83**	**125**	**225**
78	NIC1	**141**	**179**	**195**	**189**	**83**	**139**	**225**
79	NIC2	**141**	**179**	**195**	**189**	**83**	**139**	**225**
80	NIC3	**141**	**179**	**195**	**201**	**83**	**125**	**225**
81	NIC4	**141**	**179**	**162**	**189**	**83**	**125**	**225**
82	NIC5	**147**	**179**	**162**	**201**	**83**	**137**	**225**
83	NIC6	**141**	**179**	**195**	**189**	**83**	**139**	**225**
84	NIC7	**141**	**179**	**195**	**201**	**83**	**125**	**225**
85	NIC8	**141**	**179**	**195**	**201**	**83**	**125**	**225**
86	NIC9	**141**	**179**	**195**	**201**	**83**	**125**	**225**
87	NIC10	**141**	**179**	**195**	**189**	**83**	**139**	**225**
88	NIC11	**141**	**179**	**195**	**189**	**83**	**139**	**225**
89	NIC12	**141**	**179**	**195**	**201**	**83**	**125**	

90	NIC13	**141**	**179**	**195**	**189**	**83**	**139**	**225**
91	NIC14	**141**	**179**	**195**	**201**	**83**	**125**	
92	NIC15	**141**	**179**	**195**	**201**	**83**		**225**
93	NIC16	**169**	**141**	**195**	**189**	**83**	**141**	
94	NIC17	**141**	**179**		**189**	**83**	**125**	**225**
95	NIC18	**169**	**141**	**189**	**189**	**83**	**141**	**225**
96	NIC19	**169**	**141**	**189**	**189**	**83**	**141**	
97	NIC20	**169**	**141**	**189**	**189**	**83**	**141**	**225**
98	NIC21	**169**	**141**	**189**	**189**	**83**	**141**	**225**
99	NIC22	**169**	**141**	**189**	**189**	**83**	**141**	**225**
100	NIC23	**169**	**141**	**189**	**189**	**83**	**141**	**225**
101	NIC24	**169**	**141**	**189**	**189**	**83**	**141**	**225**
102	NIC25	**169**	**141**	**189**	**189**	**83**	**141**	**225**
103	NIC26	**169**	**141**	**189**	**189**	**83**	**141**	**225**
104	NIC27	**169**	**141**	**189**	**189**	**83**	**141**	**225**
105	NIC28	**169**	**141**	**189**		**83**	**141**	
106	NIC29	**141**	**179**	**162**	**189**	**83**	**125**	
107	NIC30	**141**	**179**	**195**	**189**	**83**	**139**	**225**
108	NIC31	**141**	**179**	**195**	**189**	**83**	**139**	**225**
109	NIC32	**141**	**179**	**195**	**189**	**83**	**139**	**225**
110	NIC33	**169**	**141**	**189**	**189**	**83**	**141**	**225**
111	*3D7*	***183***	**151**	**171**	**173**	**79**	**172**	***261***
112	*Dd2*	***165***	***195***	***171***	***161***	***102***	***147***	***225***
113	*HB3*	***141***	***179***	***195***	***207***	***82***	***125***	***231***
114	*7G8*	***171***	***170***	***164***		***82***	***151***	
115	*K1*	***149***	***176***	***170***	***161***	***79***	***129***	***237***
